# Supplementary material for: Mendelian randomization study of maternal influences on birthweight and future cardiometabolic risk in the HUNT cohort
Source: Nat Commun. 2020 Oct 26;11:5404. doi: 10.1038/s41467-020-19257-z (PMC7588432; doi:10.1038/s41467-020-19257-z)
Supplement: Supplementary file 1 — Supplementary Information [file 41467_2020_19257_MOESM1_ESM.pdf]

**Supplementary Note 1: Example script for running computational speed up in mother-offspring pairs**

```
######################
## Eigen decomposition ##
######################

## read in genetic relationship matrix (matrix A) and phenotype files
load("GRMoffspring_MotherOffspringPairs_fullmatrix.RData")
pheno<- read.table("mother_offspring _phenotypes.txt", header=TRUE)

## Setting parameters
y = pheno$SBP_adj_o                    # Outcome phenotype (here systolic blood pressure)
ms = pheno$full_unweightedGRS_m        # Maternal genetic risk score
cs = pheno$full_unweightedGRS_o        # Offspring genetic risk scoer
a = pheno$Age_o                        # Offspring age
s = pheno$Sex_o                        # Offspring sex
p = pheno$ParticipationRound_o         # Participation round of offspring

x <- cbind(ms,cs,a,s,p)


#Select only rows and columns from matrix to remove NA values in phenotype
```

```
select <- !is.na(y)
y_new <- y[select]
x_new <- x[select]

Anew <- A[select, select]

Eigen <- eigen(Anew) #<--New eigen decomposition of the GRM
save(Eigen_new, file = "SBP_eigenA_mother_offspring_pairs.Rdata")

###############################################
## SBP analysis with Age, Sex and Participation Round  ##
##                     Mother-Offspring pars                           ##
###############################################

rm(list=ls())
library("OpenMx")

## read in eigen file and phenotype file
load("SBP_eigenA_mother_offspring_pairs.Rdata")
pheno<- read.table("mother_offspring _phenotypes.txt ", header=TRUE)

#remove  NAs in phenotype to match eigen file
pheno_2 <- subset(pheno, !is.na(SBP_adj_o))
```

```
#Analysis : Maternal GRS - adjusted for Offspring SNPS + age +sex + participation period. All autosomal birthweight SNPs

#Generate starting values for mean of systolic blood pressure
Ybar<-mean(pheno_2$SBP_adj_o)

## Setting parameters
y = pheno$SBP_adj_o                     # Outcome phenotype (here systolic blood pressure)
ms = pheno$full_unweightedGRS_m         # Maternal genetic risk score
cs = pheno$full_unweightedGRS_o         # Offspring genetic risk scoer
a = pheno$Age_o                         # Offspring age
s = pheno$Sex_o                         # Offspring sex
p = pheno$ParticipationRound_o          # Participation round of offspring

#eigenvectors of the GRM:
yrot <- t(Eigen_new$vectors) %*% y                          #x is a column vector of the dependent variable
xrot <- t(Eigen_new$vectors) %*% cbind(1,ms,cs,a,s,p)       #x: dataframe containing a column of ones and then columns of the independent variables
datrot <- cbind(yrot,xrot,Eigen_new$values)
colnames(datrot) <- c("y","x0","ms","cs","a","s","p","eva")

# Fitting full model
TIME1<-proc.time()
```

```
MxTest_full <-mxModel("GCTA_diag",
        mxData(observed=datrot, type="raw"),        #The Yobs dataset from earlier
        mxMatrix(type="Full",nrow=1,ncol=1,free=TRUE,values=0.5,name="G"), #SNP Variance component
        mxMatrix(type="Full",nrow=1,ncol=1,free=TRUE,values=0.5,name="E"),  #Residual Variance component
        mxMatrix(type="Full",nrow=1,ncol=1, free=TRUE,values=Ybar,name="mu"), #Intercept in fixed effects part of model
        mxMatrix(type="Full",nrow=1,ncol=7, free=FALSE, labels=c("data.x0","data.ms","data.cs","data.a","data.s","data.p","data.eva"), name="defVars"),
        mxMatrix(type="Full",nrow=1,ncol=1, free=TRUE, values = 0.02, labels = "beta1", name = "b1"), #Regression coefficient
       mxMatrix(type="Full",nrow=1,ncol=1, free=TRUE, values = -0.02, labels = "beta2", name = "b2"), #Regression coefficient for child genetic score
        mxMatrix(type="Full",nrow=1,ncol=1, free=TRUE, values = 0.1, labels = "beta3", name = "b3"), #Regression coefficient for age
        mxMatrix(type="Full",nrow=1,ncol=1, free=TRUE, values = 0.1, labels = "beta4", name = "b4"), #Regression coefficient for sex
        mxMatrix(type="Full",nrow=1,ncol=1, free=TRUE, values = 0.1, labels = "beta5", name = "b5"), #Regression coefficient for participation round
        mxAlgebra(defVars[1,1]*mu + defVars[1,2]*b1 + defVars[1,3]*b2 + defVars[1,4]*b3 + defVars[1,5]*b4 + defVars[1,6]*b5, name="Ey"),
                mxAlgebra(defVars[1,7]*G + E, name="ESigma"),
                mxExpectationNormal(covariance="ESigma", means="Ey",dimnames=c("y")),
                mxFitFunctionML(rowwiseParallel=T)
)

Results<-mxRun(MxTest_full,unsafe=TRUE) #Run Model
summary(Results) #Summarize results

MxTest_sub<-mxModel("GCTA_diag_dropM",
           mxData(observed=datrot, type="raw"),     #The Yobs dataset from earlier
           mxMatrix(type="Full",nrow=1,ncol=1,free=TRUE,values=0.5,name="G"), #SNP Variance component
           mxMatrix(type="Full",nrow=1,ncol=1,free=TRUE,values=0.5,name="E"),  #Residual Variance component
```

```
    mxMatrix(type="Full",nrow=1,ncol=1, free=TRUE,values=Ybar,name="mu"), #Intercept in fixed effects part of model
    mxMatrix(type="Full",nrow=1,ncol=7, free=FALSE, labels=c("data.x0","data.ms","data.cs","data.a","data.s","data.p","data.eva"), name="defVars"),
    mxMatrix(type="Full",nrow=1,ncol=1, free=FALSE, values = 0, labels = "beta1", name = "b1"), #Regression coefficient
    mxMatrix(type="Full",nrow=1,ncol=1, free=TRUE, values = 0.1, labels = "beta2", name = "b2"), #Regression coefficient for child genetic score
    mxMatrix(type="Full",nrow=1,ncol=1, free=TRUE, values = 0.1, labels = "beta3", name = "b3"), #Regression coefficient for age
    mxMatrix(type="Full",nrow=1,ncol=1, free=TRUE, values = 0.1, labels = "beta4", name = "b4"), #Regression coefficient for sex
    mxMatrix(type="Full",nrow=1,ncol=1, free=TRUE, values = 0.1, labels = "beta5", name = "b5"), #Regression coefficient for participation round
    mxAlgebra(defVars[1,1]*mu + defVars[1,2]*b1 + defVars[1,3]*b2 + defVars[1,4]*b3 + defVars[1,5]*b4 + defVars[1,6]*b5, name="Ey"),
    mxAlgebra(defVars[1,7]*G + E, name="ESigma"),
    mxExpectationNormal(covariance="ESigma", means="Ey",dimnames=c("y")),
    mxFitFunctionML(rowwiseParallel=T)
)
Results_dropM<-mxRun(MxTest_sub,unsafe=TRUE) #Run Model
summary(Results_dropM) #Summarize results
## Setting up to compare the two models (minus two log likelihood):

#Full model:
minus2ll <- mxAlgebra(expression=GCTA_diag.fitfunction, name="minus2loglikelihood")
obj <- mxFitFunctionAlgebra("minus2loglikelihood")
model <- mxModel(model="MxTest", MxTest_full, minus2ll, obj)

base <- mxRun(model,unsafe=TRUE)
Zscore_base <- summary(base)$parameters[,5]/summary(base)$parameters[,6]
```

```
Pval_base <- 2*(1-pnorm(abs(Zscore_base),0,1))

Full <- data.frame()
Full <- rbind(Full, c(summary(base)$parameters[4,5], summary(base)$parameters[4,6], Pval_base[4]))
names(Full) <- c("Beta_cov_c", "SE_cov_c", "P_wald_cov_c")

#Model where maternal effect is constrained to zero:
minus2ll_dropM <- mxAlgebra(expression=GCTA_diag_dropM.fitfunction, name="minus2loglikelihood")
obj_dropM <- mxFitFunctionAlgebra("minus2loglikelihood")
model_dropM <- mxModel(model="MxTest", MxTest_sub, minus2ll_dropM, obj_dropM)

base_dropM <- mxRun(model_dropM,unsafe=TRUE)
Zscore_base_dropM <- summary(base_dropM)$parameters[,5]/summary(base_dropM)$parameters[,6]
Pval_base_dropM <- 2*(1-pnorm(abs(Zscore_base_dropM),0,1))

dropM <- data.frame()
dropM <- rbind(dropM, c(summary(base_dropM)$parameters[4,5], summary(base_dropM)$parameters[4,6], Pval_base_dropM[4]))
names(dropM) <- c("Beta_cov_c", "SE_cov_c", "P_wald_cov_c")

#Comparing the two models to obtain p-value:
compare <- (mxCompare(base, base_dropM))
```

**Supplementary Note 2: Details of Computational Speed Up**

Under the linear mixed model,

$$\mathbf{y} = \mathbf{Xb} + \mathbf{Wu} + \mathbf{e} \tag{1}$$

, where

$\mathbf{y}$ is an $n \times 1$ vector of phenotype scores,

$\mathbf{X}$ is the $n \times k$ design matrix for the fixed effects (covariates),

$\mathbf{b}$ is the $k \times 1$ vector of regression coefficients,

$\mathbf{W}$ is the $n \times m$ design matrix for the random effects of $m$ genetic markers,

$\mathbf{u}$ is an $m \times 1$ vector of normal i.i.d. genetic random effects, with expectation zero, and variance $\sigma_u^2$ i.e. $\mathbf{u} \sim \mathrm{N}(0, \mathbf{I}\sigma_u^2)$, and

$\mathbf{e}$ is an $n \times 1$ vector of i.i.d. residuals.

The residuals are assumed to be normally distributed, with expectation zero. Define the genomic-relatedness matrix $\mathbf{A}$ as

$$\mathbf{A} = \frac{\mathbf{WW}^T}{m} \tag{2}$$

. Conditional on $\mathbf{X}$, the variance of $\mathbf{y}$ is

$$\mathrm{var}(\mathbf{y} \mid \mathbf{X}) = \mathbf{WW}^T\sigma_u^2 + \mathbf{I}\sigma_e^2 = \mathbf{A}\sigma_g^2 + \mathbf{I}\sigma_e^2 \tag{3}$$

, where

$\sigma_g^2 = m\sigma_u^2$ is the additive-genetic variance component,

$\sigma_e^2$ is the nonshared-environmental (residual) variance component, and

$\mathbf{I}$ is an $n \times n$ identity matrix.

Consider now the eigen decomposition of $\mathbf{A}$,

$$\mathbf{A} = \mathbf{Q\Lambda Q}^T \tag{4}$$

, where

$\mathbf{Q}$ is an $n \times n$ matrix, the columns of which are the orthonormal eigenvectors of $\mathbf{A}$, and

$\mathbf{\Lambda}$ is an $n \times n$ diagonal matrix, the diagonal elements of which are the corresponding eigenvalues of $\mathbf{A}$.

Suppose we premultiply both sides of (1) by $\mathbf{Q}^T$:

$$\mathbf{Q}^T\mathbf{y} = \mathbf{Q}^T\mathbf{Xb} + \mathbf{Q}^T\mathbf{Wu} + \mathbf{Q}^T\mathbf{e} \tag{5}$$

. Then, conditional on $\mathbf{Q}^T\mathbf{X}$, the variance of $\mathbf{Q}^T\mathbf{y}$ is

$$\begin{aligned} \mathrm{var}(\mathbf{Q}^T\mathbf{y} \mid \mathbf{Q}^T\mathbf{X}) &= \mathbf{Q}^T\mathbf{WW}^T\mathbf{Q}\sigma_u^2 + \mathbf{Q}^T\mathbf{Q}\sigma_e^2 \\ &= \mathbf{Q}^T\mathbf{AQ}\sigma_g^2 + \mathbf{I}\sigma_e^2 \\ &= \mathbf{Q}^T\mathbf{Q\Lambda Q}^T\mathbf{Q}\sigma_g^2 + \mathbf{I}\sigma_e^2 \\ &= \mathbf{\Lambda}\sigma_g^2 + \mathbf{I}\sigma_e^2 \end{aligned} \tag{6}$$

Thus, the premultiplication by $\mathbf{Q}^T$has “rotated away” the dependence among the elements of $\mathbf{y}$, leaving the random effects uncorrelated. Since the random effects are assumed to be normally distributed, their lack of correlation implies their stochastic independence. Now, evaluating the likelihood of one $n$-dimensional datum has been broken up into the evaluation of $n$ 1-dimensional data, which greatly reduces the computational burden.

**Supplementary Tables 1-3:**

**Supplementary Table 1:** Results from the analysis of offspring birthweight regressed on maternal GRS*/paternal GRS* and offspring GRS. Mother-offspring pairs (*N*=7,825) and Father-offspring pairs (*N*=6,875)

| | | | **Autosomal SNPs (204 SNPs)** | | | **Autosomal SNPs with maternal effect (71 SNPs)** | | | **Autosomal SNPs with maternal effect only (31 SNPs)** | | |
|---|---|---|---|---|---|---|---|---|---|---|---|
| **Outcome** | Analysis sample | Exposure | Effect estimate | SE | p-value | Effect estimate | SE | p-value | Effect estimate | SE | p-value |
| **Offspring Birthweight (kg)** | Mother-Offspring pairs | Maternal GRS | 0.0926 | 0.0114 | 2.51E-16 | 0.0943 | 0.0114 | 1.55E-16 | 0.0804 | 0.0115 | 2.58E-12 |
| | Mother-Offspring pairs | Maternal GRS - adjusted for Offspring GRS | 0.0778 | 0.0130 | 2.13E-09 | 0.0730 | 0.0131 | 2.80E-08 | 0.0743 | 0.0131 | 1.51E-08 |
| | Mother-Offspring pairs | Offspring GRS | 0.0685 | 0.0112 | 8.02E-10 | 0.0773 | 0.0111 | 4.23E-12 | 0.0474 | 0.0112 | 2.40E-05 |
| | Mother-Offspring pairs | Offspring GRS - adjusted for Maternal GRS | 0.0321 | 0.0127 | 0.0111 | 0.0419 | 0.0128 | 1.04E-03 | 0.0121 | 0.0128 | 0.3409 |
| | Father-Offspring pairs | Paternal GRS | 0.0183 | 0.0123 | 0.1367 | 0.0369 | 0.0122 | 0.0026 | 0.0187 | 0.0122 | 0.1265 |
| | Father-Offspring pairs | Paternal GRS - adjusted for Offspring GRS | -0.0236 | 0.0139 | 0.1014 | 0.0003 | 0.0138 | 0.9762 | -0.0061 | 0.0139 | 0.6665 |
| | Father-Offspring pairs | Offspring GRS | 0.0753 | 0.0119 | 2.62E-10 | 0.0770 | 0.0119 | 1.13E-10 | 0.0478 | 0.0120 | 0.0001 |
| | Father-Offspring pairs | Offspring GRS - adjusted for Paternal GRS | 0.0857 | 0.0135 | 2.05E-10 | 0.0767 | 0.0135 | 1.21E-08 | 0.0506 | 0.0136 | 0.0002 |

The regression coefficients give the estimated expected change in offspring birthweight, per one unit (i.e. allele) increase in maternal/paternal (or offspring) genetic risk score, with or without conditioning on offspring (or maternal/paternal) genetic risk score. All analyses are adjusted for sex and measurement occasion of the offspring. Effect estimates and standard errors are standardized. P-values reflect minus two log-likelihood chi-square tests between the full model and a sub-model where the relevant parameter is fixed to zero. All p-values are two sided uncorrected for multiple testing. SNP: Single Nucleotide Polymorphism, N: number of individuals, SE: Standard Error; GRS: Genetic risk score.

*Maternal, paternal and offspring GRS were coded so that increasing dosages reflected maternal alleles associated with increased offspring birthweight based on conditional GWAS results previously published

**Supplementary Table 2:** Results from analyses stratifying mother-offspring and father-offspring pairs into two age strata

| **Young < 40 years Old Offspring *N*=12,037 (Mother-Offspring pairs; *N*=10,393 Father-Offspring pairs)** | | | | | | | | | | | |
|---|---|---|---|---|---|---|---|---|---|---|---|
| | | | Autosomal SNPs (204 SNPs) | | | Autosomal SNPs with maternal effect (71 SNPs) | | | Autosomal SNPs with only maternal effect (31 SNPs) | | |
| **Outcome** | Analysis sample | Exposure | Effect estimate | SE | p-value | Effect estimate | SE | p-value | Effect estimate | SE | p-value |
| **Offspring SBP (mmHg)** | Mother-Offspring pairs | Maternal GRS | -0.0171 | 0.0083 | 0.0389 | -0.0162 | 0.0083 | 0.0515 | -0.0236 | 0.0083 | 0.0044 |
| | Mother-Offspring pairs | Maternal GRS<br>- adjusted for Offspring GRS | -0.0155 | 0.0095 | 0.1035 | -0.0046 | 0.0095 | 0.6277 | -0.0092 | 0.0095 | 0.3349 |
| | Mother-Offspring pairs | Offspring GRS | -0.0108 | 0.0082 | 0.1877 | -0.0253 | 0.0082 | 0.0020 | -0.0332 | 0.0082 | 0.0001 |
| | Mother-Offspring pairs | Offspring GRS<br>- adjusted for Maternal GRS | -0.0033 | 0.0094 | 0.7285 | -0.0230 | 0.0094 | 0.0143 | -0.0286 | 0.0094 | 0.0025 |
| | Father-Offspring pairs | Paternal GRS | -0.0122 | 0.0090 | 0.1750 | -0.0202 | 0.0089 | 0.0244 | -0.0137 | 0.0089 | 0.1272 |
| | Father-Offspring pairs | Paternal GRS<br>- adjusted for Offspring GRS | -0.0080 | 0.0102 | 0.4352 | -0.0126 | 0.0101 | 0.2142 | -0.0025 | 0.0102 | 0.8108 |
| | Father-Offspring pairs | Offspring GRS | -0.0123 | 0.0088 | 0.1634 | -0.0215 | 0.0088 | 0.0146 | -0.0237 | 0.0089 | 0.0073 |
| | Father-Offspring pairs | Offspring GRS<br>- adjusted for Paternal GRS | -0.0085 | 0.0101 | 0.3988 | -0.0156 | 0.0100 | 0.1185 | -0.0226 | 0.0101 | 0.0265 |
| **Mid 40-60 Years Old Offspring (*N*=11,849 Mother-Offspring pairs; *N*=8,402 Father-Offspring pairs)** | | | | | | | | | | | |
| | | | Autosomal SNPs (204 SNPs) | | | Autosomal SNPs with maternal effect (71 SNPs) | | | Autosomal SNPs with only maternal effect (31 SNPs) | | |
| **Outcome** | Analysis sample | Exposure | Effect estimate | SE | p-value | Effect estimate | SE | p-value | Effect estimate | SE | p-value |
| **Offspring SBP (mmHg)** | Mother-Offspring pairs | Maternal GRS | -0.0135 | 0.0092 | 0.1405 | 0.0006 | 0.0091 | 0.9427 | -0.0077 | 0.0092 | 0.4019 |
| | Mother-Offspring pairs | Maternal GRS<br>- adjusted for Offspring GRS | -0.0059 | 0.0105 | 0.5739 | -0.0061 | 0.0087 | 0.6549 | 0.0011 | 0.0103 | 0.9125 |
| | Mother-Offspring pairs | Offspring GRS | -0.0182 | 0.0088 | 0.0399 | -0.0061 | 0.0088 | 0.4868 | -0.0179 | 0.0089 | 0.0435 |
| | Mother-Offspring pairs | Offspring GRS<br>- adjusted for Maternal GRS | -0.0156 | 0.0101 | 0.1241 | -0.0082 | 0.0100 | 0.4101 | -0.0184 | 0.0099 | 0.0657 |
| | Father-Offspring pairs | Paternal GRS | -0.0159 | 0.0109 | 0.1455 | 0.0032 | 0.0109 | 0.7689 | 0.0058 | 0.0109 | 0.5926 |
| | Father-Offspring pairs | Paternal GRS<br>- adjusted for Offspring GRS | -0.0079 | 0.0124 | 0.5228 | 0.0085 | 0.0123 | 0.4691 | 0.0179 | 0.0122 | 0.1475 |
| | Father-Offspring pairs | Offspring GRS | -0.0200 | 0.0105 | 0.0579 | -0.0071 | 0.0105 | 0.5017 | -0.0166 | 0.0105 | 0.1163 |
| | Father-Offspring pairs | Offspring GRS<br>- adjusted for Paternal GRS | -0.0164 | 0.0120 | 0.1696 | -0.0109 | 0.0119 | 0.3602 | -0.0248 | 0.0118 | 0.0386 |

The regression coefficients give the estimated expected change in offspring SBP (mmHg), per one unit (i.e. allele) increase in maternal/paternal (or offspring) genetic risk score, with or without conditioning on offspring (or maternal/paternal) genetic risk score. All analyses are adjusted for age, sex and measurement occasion of the offspring. Effect estimates and standard errors are standardized. P-values reflect minus two log-likelihood chi-square tests between the full model and a sub-model where the relevant parameter is fixed to zero. All p-values are two sided uncorrected for multiple testing. SNP: Single Nucleotide Polymorphism, N: number of individuals, SE: Standard Error; GRS: Genetic Risk Score; SBP: Systolic Blood Pressure.

NB. Maternal, paternal and offspring GRS were coded so that increasing dosages reflected maternal alleles associated with increased offspring birthweight based on conditional GWAS results previously published

**Supplementary Table 3**: Overview of numbers of offspring per parent in the analysis

| | Total number of offspring | Number of unique parents | Number of offspring in the analysis | | | | | | | |
|---|---|---|---|---|---|---|---|---|---|---|
| | | | 1 | 2 | 3 | 4 | 5 | 6 | 7 | 8 |
| Mothers | 26057 | 15261 | 8096 | 4623 | 1748 | 581 | 148 | 50 | 13 | 2 |
| Fathers | 19792 | 11867 | 6486 | 3543 | 1317 | 380 | 105 | 29 | 6 | 1 |
