# Supplementary material for: Mendelian randomization study of maternal influences on birthweight and future cardiometabolic risk in the HUNT cohort
Source: Nat Commun. 2020 Oct 26;11:5404. doi: 10.1038/s41467-020-19257-z (PMC7588432; doi:10.1038/s41467-020-19257-z)
Supplement: Supplementary file 2 — Description of Additional Supplementary Files [file 41467_2020_19257_MOESM2_ESM.pdf]

Description of Additional Supplementary Files

File Name: Supplementary Data 1

Description: Main analysis of a total of N = 26,057 mother-offspring pairs and N = 19,792 father-offspring pairs.

File Name: Supplementary Data 2

Description: SNPs used to construct genetic risk scores.
