# Supplementary material for: Mendelian randomization study of maternal influences on birthweight and future cardiometabolic risk in the HUNT cohort
Source: Nat Commun. 2020 Oct 26;11:5404. doi: 10.1038/s41467-020-19257-z (PMC7588432; doi:10.1038/s41467-020-19257-z)
Supplement: Supplementary file 5 — Reporting Summary [file 41467_2020_19257_MOESM5_ESM.pdf]

nature research

Corresponding author(s): David Evans and Gunn-Helen Moen

Last updated by author(s): Sep 21, 2020

Reporting Summary

Nature Research wishes to improve the reproducibility of the work that we publish. This form provides structure for consistency and transparency in reporting. For further information on Nature Research policies, see our Editorial Policies and the Editorial Policy Checklist.

Statistics

For all statistical analyses, confirm that the following items are present in the figure legend, table legend, main text, or Methods section.

n/a	Confirmed	
☐	☒	The exact sample size (n) for each experimental group/condition, given as a discrete number and unit of measurement
☐	☒	A statement on whether measurements were taken from distinct samples or whether the same sample was measured repeatedly
☐	☒	The statistical test(s) used AND whether they are one- or two-sided<br>*Only common tests should be described solely by name; describe more complex techniques in the Methods section.*
☐	☒	A description of all covariates tested
☐	☒	A description of any assumptions or corrections, such as tests of normality and adjustment for multiple comparisons
☐	☒	A full description of the statistical parameters including central tendency (e.g. means) or other basic estimates (e.g. regression coefficient) AND variation (e.g. standard deviation) or associated estimates of uncertainty (e.g. confidence intervals)
☐	☒	For null hypothesis testing, the test statistic (e.g. F, t, r) with confidence intervals, effect sizes, degrees of freedom and P value noted<br>*Give P values as exact values whenever suitable.*
☒	☐	For Bayesian analysis, information on the choice of priors and Markov chain Monte Carlo settings
☒	☐	For hierarchical and complex designs, identification of the appropriate level for tests and full reporting of outcomes
☒	☐	Estimates of effect sizes (e.g. Cohen's d, Pearson's r), indicating how they were calculated

Our web collection on statistics for biologists contains articles on many of the points above.

Software and code

Policy information about availability of computer code

Data collection	PLINK v1.90, Eagle2 v2.3, Minimac3 v2.0.1, KING v2.2.4, plink2 v2.00a3LM, GCTA v 1.93
Data analysis	Data analysis was performed using R version 3.6.3 and the software package OpenMx version 2.17.3 .

For manuscripts utilizing custom algorithms or software that are central to the research but not yet described in published literature, software must be made available to editors and reviewers. We strongly encourage code deposition in a community repository (e.g. GitHub). See the Nature Research guidelines for submitting code & software for further information.

Data

Policy information about availability of data

All manuscripts must include a data availability statement. This statement should provide the following information, where applicable:
- Accession codes, unique identifiers, or web links for publicly available datasets
- A list of figures that have associated raw data
- A description of any restrictions on data availability

The empirical datasets used with the HUNT study will be archived with the study and will be made available to individuals who obtain the necessary permissions from the studies' Data Access Committee. Due to privacy issues, access to individual-level data requires permission from the HUNT Study, the Medical Birth Registry of Norway and the regional committee for medical research ethics. Requirements for access to data from the HUNT Study are described at www.ntnu.edu/hunt.

Field-specific reporting

Please select the one below that is the best fit for your research. If you are not sure, read the appropriate sections before making your selection.

☒ Life sciences ☐ Behavioural & social sciences ☐ Ecological, evolutionary & environmental sciences

For a reference copy of the document with all sections, see nature.com/documents/nr-reporting-summary-flat.pdf

Life sciences study design

All studies must disclose on these points even when the disclosure is negative.

Sample size	We used all the 46,428 parent-offspring relationships available in HUNT. We did not do any sample size calculations in advance as these data were already collected and we had no way of increasing this sample size. We did however perform post hoc power calculations to check our power using the Maternal and Offspring Genetic Effects Power Calculator (https://evansgroup.di.uq.edu.au/MGPC/) which showed sufficient power for our analysis. We show in the manuscript that our power calculations indicate that we were well powered (>80% at $\alpha = 0.05$) to detect an association between maternal genetic risk score and offspring cardiometabolic risk factors responsible for as little as 0.04% of the phenotypic variance.
Data exclusions	We had a list of pre established exclusion criteria for cleaning our genotype data. These were as follows: Any individuals whose inferred sex contradicted their reported gender (N=348) as well as individuals showing high or low heterozygosity (+/- 5SD from the mean) (N=412) were removed (760 individuals in total).<br>For the phenotype data we had pre established that all phenotype values more than 4 standard deviations from the mean were removed. If the variable was not normally distributed (triglycerides, BMI and non-fasting glucose) the values were natural log transformed before removing outlying values.
Replication	No other cohort of this magnitude with same age offspring available for replication at this stage
Randomization	This is a population based cohort, and no treatments were given. We are not testing a treatment in this study.
Blinding	All data the researchers worked with were de-identified. We performed a population based study, where no treatments were given and we therefore did not have a treatment group and a control group to blind.

Reporting for specific materials, systems and methods

We require information from authors about some types of materials, experimental systems and methods used in many studies. Here, indicate whether each material, system or method listed is relevant to your study. If you are not sure if a list item applies to your research, read the appropriate section before selecting a response.

Materials & experimental systems

n/a	Involved in the study
☒	☐ Antibodies
☒	☐ Eukaryotic cell lines
☒	☐ Palaeontology and archaeology
☒	☐ Animals and other organisms
☐	☒ Human research participants
☒	☐ Clinical data
☒	☐ Dual use research of concern

Methods

n/a	Involved in the study
☒	☐ ChIP-seq
☒	☐ Flow cytometry
☒	☐ MRI-based neuroimaging

Human research participants

Policy information about studies involving human research participants

Population characteristics	For the primary analyses investigating the effect of GRS on offspring cardiometabolic traits, we had a total of 26,057 mother-offspring pairs and 19,792 father-offspring pairs. HUNT offspring were on average 40 years old, with a minimum age of 19, and a maximum age of 85 at the time of measurement used in this study. Descriptive statistics on all of the outcome variables in the two samples are presented in Table 3.
Recruitment	The Nord-Trøndelag Health Study (HUNT) is a large population-based health study of the inhabitants of Nord-Trøndelag County in central Norway that commenced in 1984.
Ethics oversight	The HUNT Study was approved by the Regional Committee for Medical and Health Research Ethics, Norway and all participants gave informed written consent (REK Central application number 2018/2488).

Note that full information on the approval of the study protocol must also be provided in the manuscript.
